# Plasma Metabolic Signature and Abnormalities in HIV-Infected Individuals on Long-Term Successful Antiretroviral Therapy

**DOI:** 10.3390/metabo9100210

**Published:** 2019-09-30

**Authors:** Hemalatha Babu, Maike Sperk, Anoop T. Ambikan, Gladys Rachel, Vinod Kumar Viswanathan, Srikanth P. Tripathy, Piotr Nowak, Luke Elizabeth Hanna, Ujjwal Neogi

**Affiliations:** 1 Department of HIV/AIDS, National Institute for Research in Tuberculosis, ICMR, Chennai 600031, India; hemalathababu89@gmail.com (H.B.); gladysrachel0292@gmail.com (G.R.); srikanth.p@nirt.res.in (S.P.T.); 2 Division of Clinical Microbiology, Department of Laboratory Medicine, Karolinska Institutet, 14152 Huddinge, Sweden; maike.sperk@ki.se (M.S.); anoop.ambikan@ki.se (A.T.A.); 3 Government Hospital of Thoracic Medicine, Tambaram Sanatorium, Chennai 600047, India; dsghtm@gmail.com; 4 Department of Medicine, Huddinge, Karolinska Institutet, 14152 Stockholm, Sweden; piotr.nowak@ki.se

**Keywords:** untargeted metabolomics, HIV/acquired immune deficiency syndrome (AIDS), targeted proteomics, antiretroviral therapy

## Abstract

Targeted metabolomics studies reported metabolic abnormalities in both treated and untreated people living with human immunodeficiency virus (HIV) (PLHIV). The present study aimed to understand the plasma metabolomic changes and predicted the risk of accelerated aging in PLHIV on long-term suppressive antiretroviral therapy (ART) in a case-control study setting and its association with the plasma proteomics biomarkers of inflammation and neurological defects. Plasma samples were obtained from PLHIV on successful long-term ART for more than five years (*n* = 22) and matched HIV-negative healthy individuals (*n* = 22, HC herein). Untargeted metabolite profiling was carried out using ultra-high-performance liquid chromatography/mass spectrometry/mass spectrometry (UHPLC/MS/MS). Plasma proteomics profiling was performed using proximity extension assay targeting 184 plasma proteins. A total of 250 metabolites differed significantly (*p* < 0.05, *q* < 0.1) between PLHIV and HC. Plasma levels of several essential amino acids except for histidine, branched-chain amino acids, and aromatic amino acids (phenylalanine, tyrosine, tryptophan) were significantly lower in PLHIV compared to HC. Machine-learning prediction of metabolite changes indicated a higher risk of inflammatory and neurological diseases in PLHIV. Metabolic abnormalities were observed in amino-acid levels, energetics, and phospholipids and complex lipids, which may reflect known differences in lipoprotein levels in PLHIV that can resemble metabolic syndrome (MetS).

## 1. Introduction

The life span of people living with human immunodeficiency virus (HIV) (PLHIV) now approaches that of HIV-negative persons because of effective combination antiretroviral therapy (cART). However, the efficacy of successful treatment depends on the lifelong intake of antiretroviral (ARV) drugs that have their side effects [1]. Also, ART shifted the spectrum of morbidity toward non-infectious complications such as diabetes, as well as cardiovascular, kidney, liver, bone-related, and neurological diseases, which appear at an earlier age in PLHIV [2]. The metabolic side effects of the ARV drugs mainly on the lipid profile has been extensively studied [3,4]. Previous studies showed that metabolic abnormalities are common in PLHIV both in untreated and treated conditions (reviewed in Reference [5]). Factors like host immune response, opportunistic infections, ART drugs, and HIV itself could be responsible for the metabolic derangement during HIV infection. Different ARV drugs have their effects on metabolic changes based on their toxicity profile like specific protease inhibitors (PIs) and nucleoside reverse transcriptase inhibitors (NRTIs), and switching drug classes to raltegravir and certain non-nucleoside reverse transcriptase inhibitors (NNRTIs) like nevirapine showed an improvement in metabolism [6].

The antiretroviral therapy program in low- and middle-income countries like India follows a public health approach. The first-line treatment regimen is reverse transcriptase inhibitor-based therapy with two nucleoside reverse transcriptase inhibitors (NRTI), tenofovir (TDF) or zidovudine (AZT) in the backbone of lamivudine (3TC), non-nucleoside inhibitors (NNRTIs) efavirenz (EFV) or nevirapine (NVP). The long-term treatment response to the first-line ART regimen was found to be good in the setting [7], even though patient monitoring was performed based on immunological and clinical definitions, unlike high-income counties where the patients are monitored virologically. Studies on the general population from India based on blood lipids, apolipoproteins, and glucose profile identified a high prevalence of individuals with blood metabolic abnormalities [8]. The prevalence of metabolic syndrome (MetS) was also higher in treatment-experienced PLHIV compared to treatment-naïve individuals [9] with the highest prevalence in the Asian region. To best of our knowledge, no complete unbiased plasma metabolic profile of PLHIV is reported from India.

The HIV–human metabolic relationship is a complex interaction made intricate even more by ART, lifestyle, and comorbidities [10]. High-throughput untargeted metabolomics best represents the molecular phenotype directly with an influence of the environment and reflects the underlying biochemical activity inside the host. Earlier untargeted metabolomics studies on biofluids [plasma and cerebrospinal fluid (CSF)] of PLHIV on short-term cART identified a signature metabolite profile that is associated with accelerated aging [11,12]. Plasma metabolomics profiles of PLHIV from the United States on more than one year on a boosted protease inhibitor (bPI) identified signature metabolites of lipid, amino-acid, and nucleotide metabolism that have distinguishing features from that of HIV-negative controls [12]. A more recent study from the Netherlands using targeted metabolites of biogenic amine, lipids, oxylipins, and oxidative stress showed minimal change after one year of ART [10]. Another untargeted metabolomics study from Spain indicated that glutaminolysis and lipoproteins are vital factors in slow immune recovery at 96 weeks of antiretroviral therapy [13]. Thus, PLHIV on suppressive ART have an increased risk of metabolomic disorders that can increase their risk of age-related diseases. The metabolic signatures in these people identified through both targeted and untargeted metabolomics were only studied well in short-term treatment, but understanding is limited in long-term therapy. Also, the environment, diet, and antiretroviral treatment regimen have an enormous impact on metabolic disease risk, and the genetic determinants of metabolism may vary across population. The studies conducted in one setting may not be reflective of another population. In our recent study, we observed that several soluble proteomics biomarkers of cancer, cardiovascular, neurological, and skeletal diseases like Eukaryotic translation initiation factor 4E (eIF4E)-binding protein 1 (4E-BP1), Adenosine Deaminase (ADA), C-C motif chemokine 23 (CCL23), T-cell surface glycoprotein CD5 (CD5), T-cell surface glycoprotein CD8 alpha chain (CD8A), Cystatin D (CST5), Matrix metalloproteinase-1 (MMP1), Neurotrophin-3 (NT3), Signaling lymphocytic activation molecule family member 1 (SLAMF1), Tumor Necrosis Factor (TNF)-related apoptosis-inducing ligand (TRAIL), and TNF-related activation-induced cytokine (TRANCE) differed significantly between PLHIV and HIV-negative controls. We, therefore, hypothesized that PLHIV on long-term successful ART are at higher risk of developing inflammatory diseases leading to inflammaging [14]. The present exploratory study aims to understand the metabolomic changes and associated risk of accelerated aging in PLHIV on a long-term suppressive RTI-based ART regimen. We also examined the relationship between plasma markers of inflammation and neurological defects and identified a distinct metabolite signature in PLHIV as compared to age- and lifestyle-matched healthy controls. To the best of our knowledge, this is the first comparative study with complete and unbiased profiling of circulating metabolites in PLHIV from the country.

## 2. Results

### 2.1. Cohort Characteristics

The cohort characteristics are presented in Table 1. The participants were screened from a larger cohort of 553 individuals attending a tertiary care ART Center for routine standard of care from the southern part of India. The complete cohort characteristics are presented elsewhere [14]. All the PLHIV were initiated on treatment with very low CD4^+^ T-cell count, but all of them had higher CD4^+^ T-cell count at sampling. The leucocyte telomere length was significantly shorter in PLHIV than matched HIV-negative healthy individuals (HC). The median [interquartile range (IQR)] latent HIV-1 reservoir quantified by total HIV-1 DNA copy numbers was 2.78 (2.58–3.01) copies/10^6^ peripheral blood mononuclear cell (PBMCs). Among the PLHIV, 59% (13/22) were on AZT/3TC/NVP and 41% (9/22) were on TDF/3TC/EFV, which is the first line of therapy in the country, and they did not have any evidence of treatment failure.

### 2.2. Metabolite Summary

**Figure 1:**
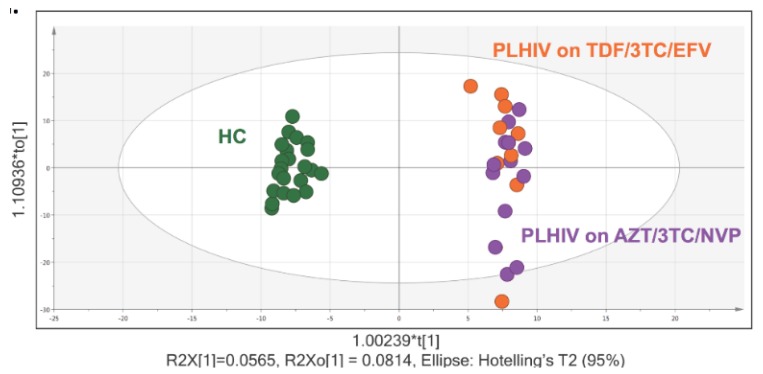
Orthogonal partial least square discriminant analysis (OPLS-DA) and metabolite summary: (a) OPLS plot using 1114 biochemicals, of which 901 compounds were of known identity (named biochemicals) and 213 compounds of unknown structural identity (unnamed compounds) identified group clustering of human immunodeficiency virus (HIV)-negative control (green) and people living with HIV (PLHIV) on Tenofovir/Lamivudine/Efavirenz (TDF/3TC/EFV) (orange) and PLHIV on Zidovudine/Lamivudine/Nevirapine (AZT/3TC/NVP) (purple).

We identified 1114 bio-chemicals, of which 901 compounds were of known identity (named biochemicals) and 213 compounds of unknown structural identity (unnamed compounds). To analyze variations between the two groups, supervised analysis with orthogonal partial least square discriminant analysis (OPLS-DA) and unsupervised analysis with principal component analysis (PCA), which transforms a large number of metabolic variables into a smaller number of orthogonal variables, were performed. A clear separation between PLHIV and HC groups was seen, with more clustering in HC but scattered in PLHIV, and more association in the Tenofovir/Lamivudine/Efavirenz (TLE) regimen in OPLS-DA analysis (Figure 1). A similar separation between the two groups was also observed in the PCA analysis (Figure S1, Supplementary Materials).

**Figure 2:**
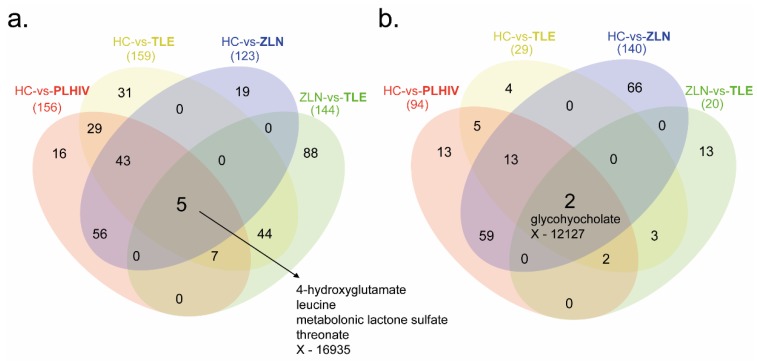
Metabolite summary. Four different comparative analyses were performed on PLHIV compared to healthy control (HC vs. PLHIV), patients on Tenofovir/Lamivudine/Efavirenz (TLE) compared to HC (HC vs. TLE), patients on Zidovudine/Lamivudine/Nevirapine (ZLN) compared to HC (HC vs. ZLN), and patients on ZLN compared to patients on TLE (ZLN vs. TLE). (**a**) Number of metabolites that were significantly (*p* < 0.05, *q* < 0.1) lower in PLHIV, TLE, and ZLN (marked in bold) compared to HC and patients on ZLN (marked in bold) compared to patients on TLE. (**b**) Number of metabolites that were significantly (*p* < 0.05, *q* < 0.1) higher in PLHIV, TLE, and ZLN (marked in bold) compared to HC and patients on ZLN (marked in bold) compared to patients on TLE.

The *t*-tests identified 250 significantly different metabolite levels (*p* < 0.05, false discovery rate (FDR, *q*) < 0.10) between PLHIV and HC. Among the metabolites, 156 were lower (Figure 2a) while 94 were higher in PLHIV compared to HC (Figure 2b). While underpowered, within the PLHIV group, there were 164 significantly different metabolites [(144 lower and 20 higher in TLE compared to Zidovudine/Lamivudine/Nevirapine (ZLN)] between the individuals on TLE and ZLN treatment regimens (Figure 2b). There were five metabolites that were lower in PLHIV compared to HC and also lower in patients with TLE compared to the ZLN groups (4-hydroxyglutamate, leucine, metabolonic lactone sulfate, threonate, and X-16935, where X-16935 is a novel compound). All the significant comparisons presented in Venn diagrams are given in the supplementary data file 1.

**Figure 3:**
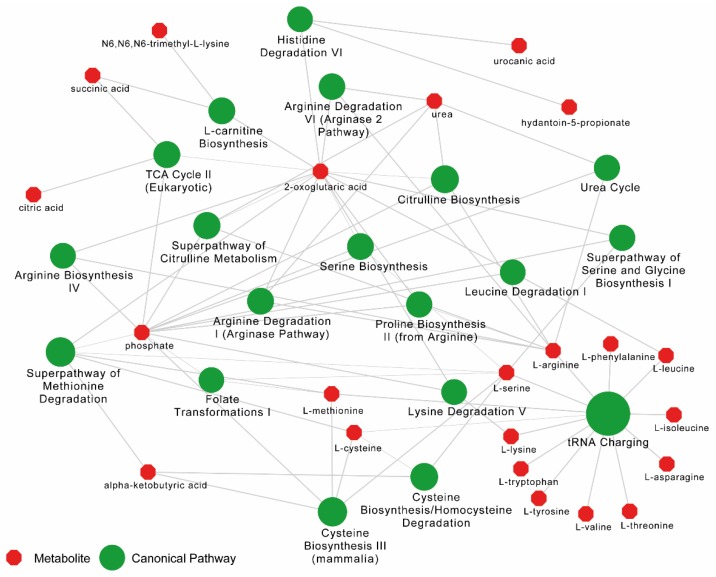
Pathway enrichment analysis results from Ingenuity Pathway Analysis (IPA) for metabolome data between PLHIV and HC. Top 20 canonical pathways (in green) are shown. Bubble sizes are relative to *p*-values. The metabolites (in red) related to each pathway are linked with a continuous gray line.

The pathway enrichment analysis was carried out in ingenuity pathway analysis (IPA) (Qiagen, Germany) with metabolites with the Human Metabolome Database (HMBD) IDs (*n* = 228 mapped). The functional annotation by canonical pathways associated with differential levels of metabolites revealed that top 20 pathways were mainly related to the differential level of amino acids (Figure 3).

### 2.3. Disease and Biofunction Predicted by Ingenuity Pathway Analysis (IPA)

**Figure 4:**
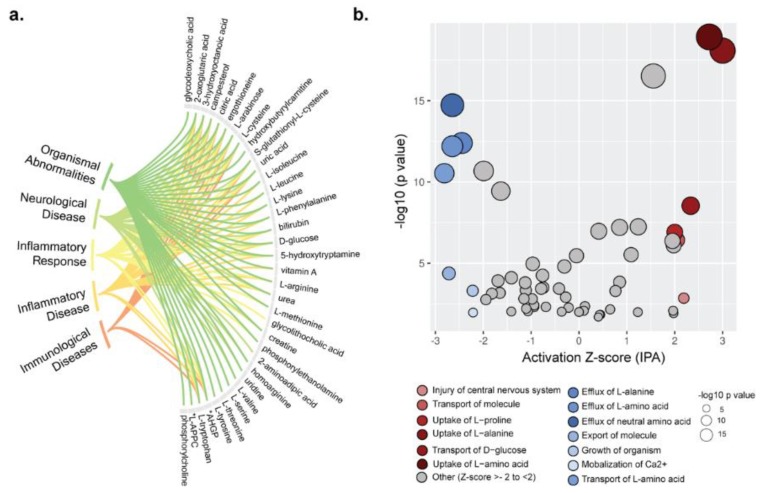
Metabolome annotation and biofunction analysis. (**a**) Metabolome annotation results obtained from ingenuity pathway analysis (IPA). The figure visualizes the most significant biological functions (*p* < 0.05) and corresponding metabolites. (**b**) Pathway enrichment analysis results from IPA for metabolome data. IPA generated activation *z*-scores in the *x*-axis and *p*-values in the *y*-axis. Bubble sizes are relative to *p*-values. A negative *z*-score implies significant downregulation of the pathway and vice versa.

To infer the possible biological impact associated with the metabolic changes in PLHIV, we used IPA on those metabolites with HMBD IDs (*n* = 228 mapped). IPA predicted effects on disease and biofunctions related to immunological disease, inflammatory disease and inflammatory response, neurological disease, and organismal injury and abnormalities (*p* < 0.05) (Figure 4a). Prediction of activation or inhibition of a disease or biofunction in PLHIV was calculated as a negative or positive *z*-score. There was an increase in the uptake of specific amino acids including l-alanine and l-proline, as well as transport of d-glucose, injury of the central nervous system, and transport of molecules (*p* < 0.001) (Figure 4b).

### 2.4. Identification of Potential Biomarkers

**Figure 5:**
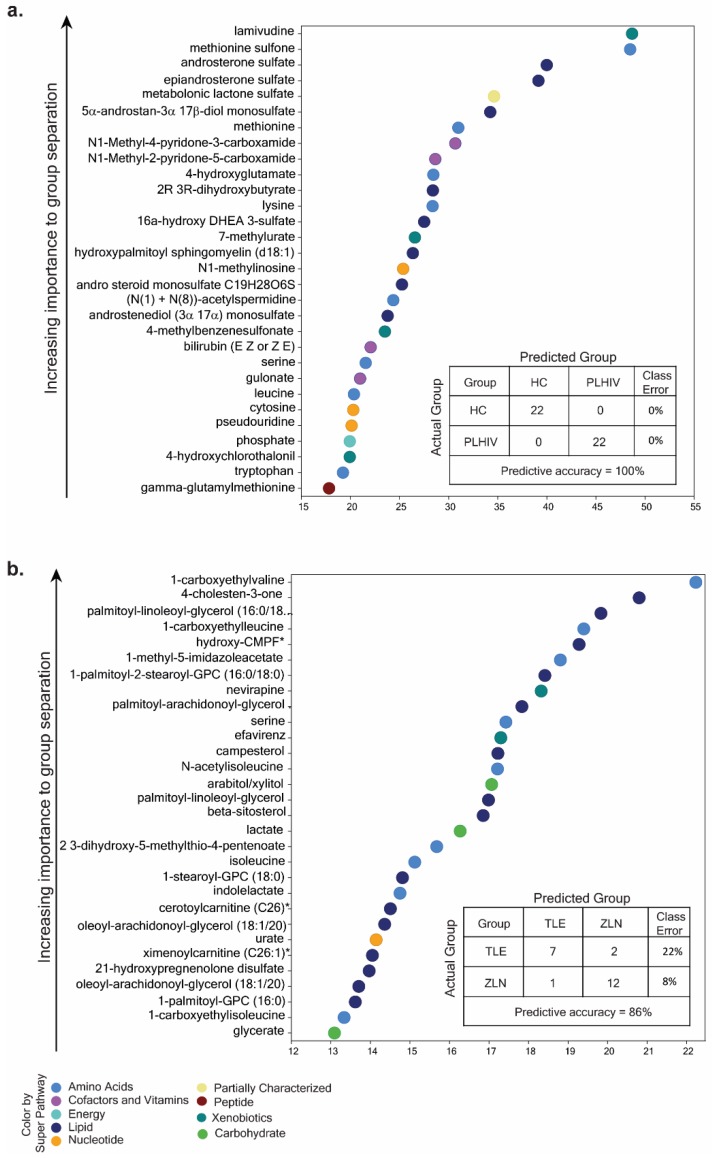
(**a**) Random forest (RF) analysis of named biochemicals resulted in predictive accuracies of 100% for HC vs. PLHIV. The biochemical importance plots display the top 30 metabolites which most strongly contribute to the groups’ separation for HC vs. PLHIV based on amino-acid metabolism, lipid metabolism, energy metabolism, co-factors and vitamins, peptides, and xenobiotics, as indicated in different colors in the legend. (**b**) The RF analysis of named biochemicals resulted in predictive accuracies of 86% for PLHIV-TLE vs. PLHIV-ZLN. The biochemical importance plots display the top 30 metabolites which most strongly contribute to the groups’ separation based on amino-acid metabolism, carbohydrate metabolism, lipid metabolism, nucleotide metabolism, and xenobiotics.

To identify potential biomarkers of interest among the significant biochemicals, random forest (RF) analysis was performed using the two groups (HC vs. PLHIV) and sub-groups (PLHIV-TLE vs. PLHIV-ZLN) to examine the effect of treatment regimen on biochemicals. We performed the analysis either with only identified named biochemicals (*n* = 901) or including all biochemicals (*n* = 1114). The RF analysis of named biochemicals resulted in predictive accuracies of 100% for HC vs. PLHIV (Figure 5a) and 86% for PLHIV-TLE vs. PLHIV-ZLN (Figure 5b). While including all the metabolites, the predictive accuracy remained unchanged for HC vs. PLHIV but increased to 91% for PLHIV-TLE vs. PLHIV-ZLN (Figure S2, Supplementary Materials). The biochemical importance plots display the top 30 metabolites which most strongly contribute to the groups’ separation for HC vs. PLHIV (Figure 5) and PLHIV-TLE vs. PLHIV-ZLN (Figure 5b). Among the top 30 metabolites, most differentiating biochemicals in HC vs. PLHIV were involved in amino-acid metabolism (8/30) and lipid metabolism (8/30). Two biochemicals that stood out as particularly interesting were methionine sulfone and metabolomic lactone sulfate (Figure 5a). In PLHIV-TLE vs. PLHIV-ZLN, half of the biochemicals were part of lipid metabolism, while 30% (9/30) were part of amino-acid metabolism.

**Figure 6:**
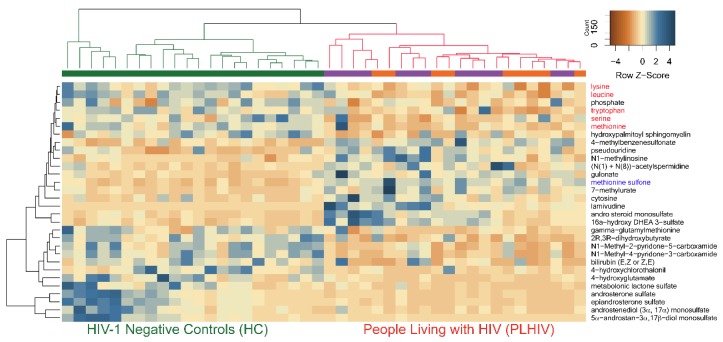
The hierarchical clustering of all the samples (HC and PLHIV) indicated negative *z*-scores for lysine, leucine, methionine tryptophan, and serine (marked in red), indicating lower levels in PLHIV compared to HC; however, methionine sulfone (marked in blue) had a higher level with positive *z*-scores in PLHIV.

The hierarchical clustering of all the samples (HC and PLHIV) indicated negative *z*-scores for lysine, leucine, methionine, tryptophan, and serine, indicating the lower level in PLHIV compared to HC; however, methionine sulfone had a higher level with positive *z*-scores in PLHIV (Figure 6). Despite the smaller sample size, these 30 metabolites were powered enough for group separation between PLHIV-TLE vs. PLHIV-ZLN, as observed in PCA (Figure S3).

### 2.5. Altered Amino-Acid Metabolism and Kynurenine-to-Tryptophan (K/T) Ratio in PLHIV

**Figure 7:**
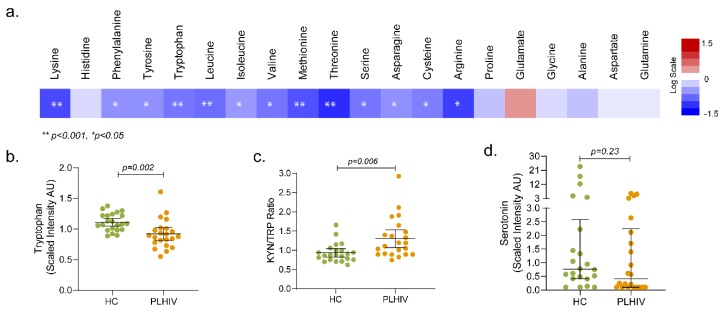
Metabolic pathways associated with HIV-1 infection following long-term therapy (**a**) Altered amino-acid metabolism. Amino acids (*n* = 20) that were significantly lower (in blue) or higher (in red) in PLHIV compared to HC. The level of tryptophan (**b**), Kyn/Trp ratio (**c**), and serotonin (**d**).

Amino acids play an essential role both as regulators in several metabolic pathways and as primary substrates. Lower levels of all essential amino acids (EAA; lysine, phenylalanine, tryptophan, leucine, isoleucine, valine, methionine, and threonine) except histidine were observed in PLHIV compared to HC, which include the branched-chain amino acids (BCAA; leucine, isoleucine, and valine) and aromatic amino acids (AAA; phenylalanine, tyrosine, and tryptophan) (Figure 7a).

Tryptophan levels were significantly reduced in PLHIV compared to HC (Figure 7b); however, no statistically significant difference was observed in kynurenine levels. Despite that, the kynurenine-to-tryptophan (K/T) ratio was significantly increased in PLHIV (Figure 7c). Although there was no statistical significance, 50% of the PLHIV had lower serotonin than the lower quartile (Figure 7d). These data indicate that the PLHIV on ART maintain certain levels of systemic inflammation.

### 2.6. Altered Energy Metabolism

**Figure 8:**
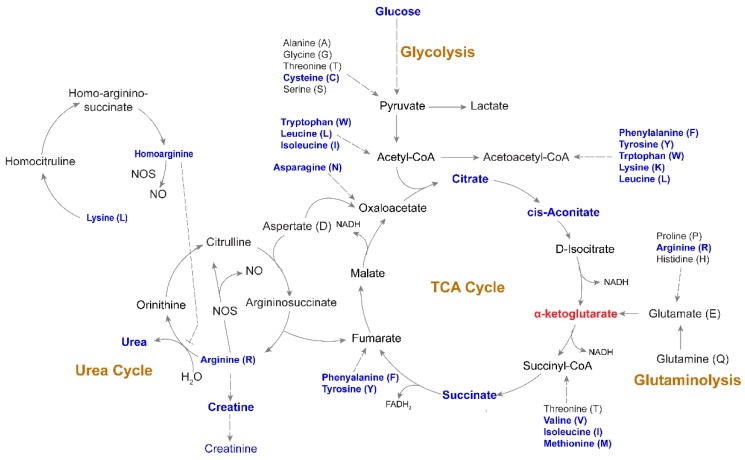
Altered metabolic pathways associated with HIV-1 infection following long-term therapy Schematic map of tricarboxylic acid (TCA) cycle, glycolysis, urea cycle, and glutaminolysis. Blue indicates a significantly (*p* < 0.05) lower level of metabolites in PLHIV compared to HC. α-ketoglutarate was the only metabolite that had a significantly higher level (marked in red) in PLHIV compared to HC.

Glucose levels were significantly lower in the PLHIV compared to HC. Via a series of enzyme-catalyzed steps, glucose is metabolized to pyruvate which can either be oxidized in the mitochondria to enter the tricarboxylic acid cycle TCA cycle as acetyl Coenzyme A (CoA) or reduced to lactate by lactate dehydrogenase (LDH). Interestingly, the lower level of glucose did not result in lower pyruvate or lactate in the PLHIV group. The mitochondrial TCA cycle links the catabolism of carbohydrates, lipids, and some amino acids to ATP production via the supply of reducing equivalents through oxidative phosphorylation. In the current study, citrate, aconitate, and succinate were lower in PLHIV compared to HC (Figure 8). TCA cycle carbon can be supplemented by the glutaminolysis process, in which glutamine is converted to glutamate, then to α-ketoglutarate, which was higher in PLHIV subjects. It is possible that glutaminolysis supported α-ketoglutarate, as neither glutamate nor glutamine was altered in PLHIV. Likely because of decreased amino-acid levels, levels of urea cycle metabolites, including the amino-acid arginine and urea, were lower in PLHIV samples (Figure 8). Homoarginine was also lower in the PLHIV group, but citrulline was not different. These data suggest that PLHIV would produce lower nitric oxide. However, this would need to be assessed in a cell-based assay.

### 2.7. Changes in Phospholipids, Ceramides, and Other Complex Lipids

Phospholipids are synthesized from diacylglycerols and polar head groups such as choline or ethanolamine and circulate in the plasma as constituents of lipoproteins synthesized mostly in the liver. In our study, several phosphatidylcholines (PC; e.g., 1-stearoyl-2-oleoyl-sn-glycero-3-phosphocholine (18:0/18:1)) were higher in the PLHIV group compared to the HC (Figure S4). GPC-lysophospholipids (e.g., 1-lignoceroyl-GPC (24:0)) were also higher in the PLHIV group. There were significant changes in other complex lipids in the plasma of the PLHIV, which may be markers of inflammation, immune cell function, and oxidative stress. The ceramides (e.g., *N*-palmitoyl-sphingosine (d18:1/16:0)) were higher in PLHIV compared to HC samples. Sphingomyelins (e.g., sphingomyelin (d18:2/18:1)) and dihydrosphingomyelins (e.g., palmitoyl dihydrosphingomyelin (d18:0/16:0)) were lower in the PLHIV group compared to HC.

### 2.8. Plasma Proteomic Profile and Its Correlation with Metabolite

**Figure 9:**
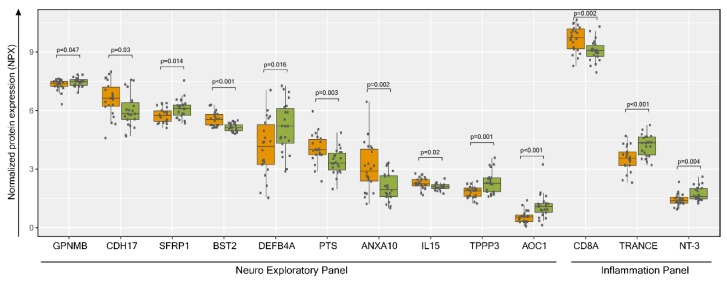
Plasma proteomics profiling and protein metabolite interactions. Normalized protein expression (NPX) visualization of significantly enriched proteins (*p* < 0.05) from Welch’s two-sample *t*-test between HC (green) and PLHIV (orange) groups. GPNMB: Transmembrane glycoprotein NMB (GPNMB), CDH17: cadherin-17, SFRP1: secreted frizzled-related protein 1, BST2: bone marrow stromal antigen 2, DEFB4A: defensin beta 4, PTS: 6-pyruvoyltetrahydropterin synthase, ANXA10: annexin A 10, IL15: Interleukin 15, TPPP3: tubulin polymerization-promoting protein family member 3, AOC1: amiloride-sensitive amine oxidase (copper-containing), CD8A: T-cell surface glycoprotein CD8 alpha chain, TRANCE: Tumor Necrosis Factor (TNF)-related activation-induced cytokine, NT-3: neurotrophin-3.

The plasma metabolomics profile indicated a higher risk of inflammatory and neurological diseases in PLHIV. We next performed plasma proteome profiling targeting 184 proteins in two Olink^®^ panels: inflammation targeting inflammation-related proteins and neuro-exploratory, targeting neurology-related diseases, and biological processes such as axon development, neurogenesis, and synapse assembly. Among the proteins analyzed, 11 from the neuro-exploratory and three from the inflammation panel were significantly different between the groups (Figure 9).

**Figure 10:**
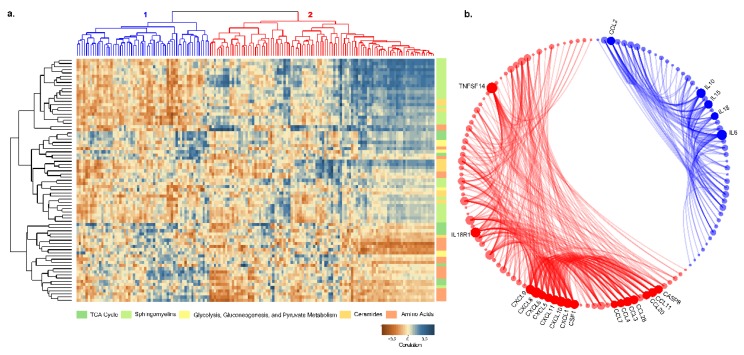
(**a**) Pearson correlation coefficient visualization and hierarchical clustering between metabolites and proteins. Metabolites belonging to various categories were plotted in rows and proteins in columns. (**b**) Protein–protein interaction results between two clusters of proteins from correlation clustering. Blue elements are cluster-1 proteins, and red elements are cluster-2 proteins. Bubble size is relative to the number of interactions, and each ribbon implies an interaction.

Next, we performed a correlation analysis between plasma proteins and metabolites in five distinct pathways in PLHIV. There were two different clusters of proteins while correlating with the metabolites (Figure 10a). In cluster 1, most of the proteins were positively correlated with amino acids, while cluster 2 proteins were positively co-related with ceramides and sphingomyelins. While performing the functional links between proteins in clusters 1 and 2 in STRING-protein interaction database, a strong association between the proteins was observed. Most of the interleukins were in cluster 1, while CXC chemokine and C–C motif chemokine were in cluster 2 (Figure 10b).

## 3. Discussion

In the present study, we found a pattern of metabolic abnormalities in several biological pathways involved in amino-acid metabolism, energy metabolism, urea, and TCA cycle, with changes in ceramides, phospholipids, and other complex lipids in PLHIV in spite of them being on suppressive cART for several years. The untargeted metabolomics approach identified 1114 metabolites including 213 unnamed biochemicals in PLHIV, which provides an unbiased picture of metabolite abnormalities. The predicted effects of these deranged metabolites on disease and biofunctions that are related to immunological, inflammatory, and neurological disorders were significant in PLHIV. Collective analysis of plasma proteomic profiles with the metabolic profile in this cohort and the proteomics profile in the larger cohort recently described by us [14] indicated the persistence of systemic residual inflammation along with a higher risk of age-associated immunological, inflammatory, and neurological diseases, organismal injury, and abnormalities in PLHIV.

Chronic HIV infection and resultant inflammation continuously activate the host immune system. Consequently, there is a metabolic imbalance in the host immune system, which can be aggravated by the ART toxicities. Among the changes in the metabolomic processes, distinct changes were observed in amino-acid metabolism and differences in energetics. The machine learning algorithm predicted that the PLHIV are at risk for developing neurological disease and organismal injury and abnormalities in addition to immunological and inflammatory diseases. Antiretroviral drugs like zidovudine (AZT), which is part of the first-line ART in India, induce mitochondrial dysfunction by inhibition of DNA pol-γ activity [15] that can cause oxidative stress. Several studies reported a decrease in antioxidant levels in PLHIV on ART compared to the treatment naïve HIV-positive and HIV-negative controls [10,16]. In the RF analysis, the top two metabolites were methionine sulfone and metabolomic lactone sulfate. Higher levels of methionine sulfone (an irreversible oxidation product of methionine) were identified in PLHIV on ART compared to HIV-negative controls. Also, specific ceramides are increased in PLHIV and are known to track 4-hydroxynonenal (4-HNE) [17]. Higher levels of methionine sulfone and ceramides are indicators of increased oxidative stress, which was observed in earlier studies [5]. A recent study from the Netherlands that analyzed targeted metabolites of biogenic amine, lipids, oxylipins, and oxidative stress showed minimal change after one year of ART [10]. Our study is, therefore, indicative that, even after long-term suppressive therapy, persistent chronic oxidative stress exists in PLHIV. If not controlled, oxidative stress can be responsible for the induction of age-related chronic and degenerative diseases like cardiovascular diseases (CVDs) [18], several neurological disorders including depression and memory loss [19], rheumatoid arthritis, kidney diseases, etc. [20]. It may facilitate and accelerate aging in PLHIV.

Neurocognitive impairment and HIV-associated neurological disorders (HANDs) were reported in PLHIV displaying different symptoms, including HIV-associated dementia (HAD) [21]. an increase in K/T ratio and a lower level of serotonin in more than half of the PLHIV were observed in our study. Several plasma proteomics markers related to neurological diseases were also altered in these patients. Transmembrane glycoprotein NMB (GPNMB), secreted frizzled-related protein 1 (SFRP1), defensin beta 4 (DEFB4A), tubulin polymerization-promoting protein family member 3 (TPPP3), amiloride-sensitive amine oxidase (copper-containing), (AOC1) and CD8A were decreased in PLHIV compared to HC, while cadherin-17 (CDH17), bone marrow stromal antigen 2 (BST2), 6-pyruvoyltetrahydropterin synthase (PTS), Tumor Necrosis Factor (TNF)-related activation-induced cytokine (TRANCE), neurotrophin-3 (NT-3), and annexin A 10 (ANXA10) were increased in PLHIV compared to HC. The role of each protein during HIV infection, and sometimes even protein function itself are not always clear. For instance, DEFB4A, also known as human beta-defensin 2 (hBD-2) or skin-antimicrobial peptide (SAP1), is an antimicrobial peptide with activity against both Gram-positive and Gram-negative bacteria. Recombinant hBD-2 (and hBD-3) was shown to inactivate HIV-1 in vitro directly. Contrarily, elevated hBD-2 levels in connection with microbial translocation in the gastrointestinal (GI) tract in HIV-infected individuals or genital infections in HIV-infected women may also promote local inflammation and viral transmission [22]. Levels of hBD-2 were, however, lower in our cohort of PLHIV compared to HC, which might indicate reduced anti-HIV-1 activity of defensins in long-term treated HIV-infected patients. Whether that is due to lower viral replication or impaired anti-HIV defense mechanisms needs further investigation. However, interferon-inducible antiviral factor BST-2 (tetherin) that inhibits HIV-1 viruses was elevated in the PLHIV group [23,24]. Of note, elevated BST2 is a plasma marker of colorectal carcinoma [25].

PLHIV exhibited distinct changes in plasma EAA levels when compared to HC. Out of nine EAAs, the levels of eight were lower in PLHIV, essentiality defining that all the EAAs must be derived from exogenous sources, mainly via diet. Lower levels of EAAs in PLHIV, thus, indicate that either these subjects were suffering from protein malnutrition (dietary intake below requirement), or the intestinal absorption of these amino acids was compromised when dietary protein intake was optimal to sustain the plasma amino-acid balance. Although no nutritional protein intake data for the study groups are available, BMI index and the food habits from the study cohorts indicate no tangible differences between the two study groups; thus, the plausible involvement of low protein intake as the causative factor in plasma amino-acid imbalance is minimal. Therefore, impaired uptake of these amino acids in gut epithelia could be a potential causative factor in relation to chronic gut inflammation in HIV following ART [26] or cART-mediated inhibition of amino-acid uptake by enterocytes in systemic metabolic disorder. This requires further in-depth investigation to mechanistically establish as to why PLHIV suffer from such an EAA imbalance.

Amino acids further correlate with a cluster of proteins that includes several interleukins (ILs), and these ILs are strongly associated with each other. Of note, only IL-15 showed significantly different levels in PLHIV compared to HC; IL-6, IL-10, and IL-18 did not. Several earlier studies were aimed at deciphering the roles of proinflammatory cytokines, for example, IL-6, in muscle turnover/muscle wasting. Both muscle synthesis and breakdown might be negatively affected by IL-6, but the severity of the impact is relatively higher on the former, resulting in increased net muscle breakdown. Also, IL-6 might increase AA demands of other tissues than muscle resulting in decreased AA levels in plasma. Although the exact mechanisms remain unclear, it can be concluded that cytokines can initiate essential changes in secondary mediators and clinical complications, causing muscle wasting [27,28]. IL-18, as another example of the interplay between cytokines and cell metabolism, was described to upregulate nutrient transporters in natural killer (NK) cells causing metabolic changes to support NK cell proliferation [29]. Therefore, the role of cytokines in AA metabolism, both in healthy individuals and in states of inflammatory diseases, remains unclear, and, from this study, it was found to be changed in HIV infection and conditions of antiretroviral therapy.

Another cluster of proteins, including several chemokines, is correlated with ceramides and sphingomyelins both of which belong to the class of sphingolipids. Indeed, sphingolipids were described as lipid mediators that are involved in diverse cellular functions, including immune cell trafficking and inflammation. Ceramides are central sphingolipid metabolites that are associated with proapoptotic and inflammatory signaling. As such, they induce inflammatory responses and regulate immune cell functions [30,31]. The exact association of cytokines and chemokines with the metabolites still needs to be elucidated.

The present study has some limitations that are worth mentioning. Firstly, the sample size was relatively small. The traditional approaches are not easily transferable due to the top-down hypothesis-free characteristic (untargeted approach) of -omics studies. However, our analysis showed that, even in a small number of patients, untargeted metabolomics has the power to separate the groups while using the top 30 metabolites. Secondly, as adherence is one of the biggest problems in low- and middle-income countries (LMICs) and the PLHIV included in the study were one of the best adherent groups of HIV-infected individuals, the result may not reflect what was seen in non-adherent patients.

## 4. Materials and Methods

### 4.1. Study Cohorts

The present study included HIV-1 positive individuals with successful long-term ART for more than five years (*n* = 22, PLHIV herein) and age-, sex-, and lifestyle-matched HIV-1 negative healthy individual without any chronic illness (*n* = 22, HC herein). The participants were screened from a larger cohort of 553 individuals attending a tertiary care ART Center for routine standard of care at the Government Hospital for Thoracic Medicine (GHTM), Chennai, India. The inclusion criteria were HIV-1 positive status, stable CD4 counts for the past 24 months, and >90% treatment adherence. Participants were excluded if there were pregnant women, or patients who had any opportunistic infection or major psychiatric illness, with co-infections like active tuberculosis, and co-morbidities like diabetes mellitus and obesity, with evidence of cardiovascular diseases or any chronic illness, illicit drug usage, and alcohol consumption. HIV-negative samples were collected from age-, sex-, food habit-, and lifestyle-matched healthy individuals from in and around Chennai, India. A single non-fasting blood sample was collected. The study was approved by the Institutional Ethics Committee of the National Institute for Research in Tuberculosis (NIRT IEC No: 2015023 and TRC IEC No: 2011001) and Institutional Review Board Committee of Government Hospital for Thoracic Medicine (GHTM-27102015). Informed consent was obtained from all participants according to the guidelines of the institutional ethics committee.

### 4.2. Sample Preparation and Ultra-High-Performance Liquid Chromatography/Mass Spectrometry/Mass Spectrometry (UHPLC/MS/MS)

Untargeted metabolite profiling was carried out by Metabolon Inc. (Durham, NC, USA) using ultra-high-performance liquid chromatography/mass spectrometry/mass spectrometry (UHPLC/MS/MS). Plasma (100 μL) was mixed with methanol to recover chemically diverse metabolites after precipitating proteins. The methanol extract was divided into four fractions: two for analysis by two separate reverse-phase (RP)/UPLC/MS/MS methods with positive ion mode electrospray ionization (ESI), one for analysis by RP/UPLC/MS/MS with negative ion mode ESI, and one for analysis by HILIC/UPLC/MS/MS with negative ion mode ESI. The MS analysis alternated between MS and data-dependent MS^n^ scans using dynamic exclusion. A pooled sample was created by taking a small aliquot from each of the samples, which served as technical replicates in the assay, whereas pure water samples served as a process blank, and a cocktail of quality control (QC) standards (Metabolon) was spiked into every standard sample to identify the instrument variability. The instrument variability determined by calculating the median relative standard deviation (RSD) for the internal standards was 4%. The samples from the two groups were randomized across the platforms, and internal standards and process blanks were added to each sample prior to injection into the mass spectrometers

### 4.3. Data Extraction, Compound Identification, and Quantification

The raw data extraction, peak identification, and QC process were performed using Metabolon proprietary hardware and software. The metabolites were identified using a proprietary in-house library based on standards that contained the retention time/index (RI), mass to charge ratio (*m/z*), and chromatographic data (including MS/MS spectral data) on molecules present in the library. Additional mass spectral entries were created for structurally unnamed biochemicals, which were identified by their recurrent nature (both chromatographic and mass spectral). Peaks were quantified using the area under the curve. The biochemical data were normalized for the volume of plasma.

### 4.4. Plasma Proteomics Profiling

Plasma proteomic profiling was performed using proximity extension assay (PEA) technology (Olink Bioscience AB, Uppsala, Sweden). We selected the Olink^®^ inflammation panel that includes 92 inflammation-related protein biomarkers and a neuro-exploratory panel that consists of 92 proteins for neurology-related diseases. The details of the markers available, their analytical range, and their lower detection limit in each panel are available at https://www.olink.com/resources-support/document-download-center/.

### 4.5. Statistical and Bioinformatics Analysis

Log-transformed and volume normalized data were used for standard statistical analysis. Feature ranking process using random forest algorithm was executed using R package randomForest. Welch’s two-sample *t*-test and correlation analysis were performed using R package stats. Volcano plots and heatmaps were generated using R package ggplot and gplots, respectively. CIRCOS software package was used to create a circos plot, and the bubble circos plot was created using the R packages graph and igraph. A *p*-value < 0.05 and a false discovery rate (*q*) < 0.10 was considered as significant. Metabolite and pathway networks were created with Cytoscape ver 3.7.1 [32].

## 5. Conclusions

In conclusion, the plasma metabolome of PLHIV on ART was significantly different from that of age-, sex-, and lifestyle-matched HIV-negative controls despite a median duration of nine years of treatment. Metabolic abnormalities were observed in amino-acid levels, energetics, and phospholipids and complex lipids, which may reflect known differences in lipoprotein levels in PLHIV that can resemble metabolic syndrome (MetS). Chronic metabolic disorder and an indication of oxidative stress can contribute to the development of age-related diseases and can accelerate the aging process in PLHIV. Combining the data from our recent study on plasma biomarkers of inflammation [14] and the present metabolic profile indicates that that PLHIVs on long-term therapy are at higher risk of inflammaging and age-related inflammatory diseases. With the advancement of the high-throughput metabolomics platforms and the use of less toxic drugs in clinical practice, further studies are under way to identify the metabolic network that is affected by HIV infection and/or antiretroviral treatment which could be of interest for therapeutic intervention in HIV-infected individuals.

## Figures and Tables

**Table 1:** Participant clinical and demographic data.

Parameter	HIV-1 Negative Healthy Control	PLHIV on Therapy	*p*-Values
Number	22	22	
Age in years, mean (95% CI)	45 (43–47)	45 (43–48)	0.91
Sex, Female, *n* (%)	10 (45%)	9 (41%)	0.99
Telomere length, Mean (95% CI) ^1^	5.9 (4.3–7.2)	2.3 (1.6–2.9)	<0.001
Nadir CD4 count, median (IQR))	-	229 (176–318)	-
CD4 count at sampling, median (IQR)	-	624 (524–746)	-
CD8 count at sampling, median (IQR)	-	757 (665–882)	-
Viral load, copies/mL	-	<150	-
CD4:CD8 ratio, median (IQR)	-	0.8 (0.58–1.02)	-
Years on therapy, median (IQR)	-	9 (6–10)	-
Reservoir, log_10_ copies/10^6^ cells, median (IQR)	-	2.78 (2.58–3.01)	-
Treatment regimen AZT/3TC/NVP TDF/3TC/EFV	-	13 (59%) 9 (41%)	-

^1^ Leucocyte, on each chromosome end, IQR: Interquartile range, CI: Confidence interval, AZT/3TC/NVP: Zidovudine/Lamivudine/Nevirapine, TDF/3TC/EFV: Tenofovir/Lamivudine/Efavirenz.

## Supplementary Materials

The following are available online at https://www.mdpi.com/2218-1989/9/10/210/s1: Figure S1: Principal component analysis (PCA). The PCA identified separation between HC and PLHIV samples with PLHIV samples separating to the north and east of HC samples. There was also some separation of samples in the PLHIV group based on treatment type: TDF/3TC/EFV (TLE) and AZT/3TC/NVP (ZLN); Figure S2: Random forest analysis using all metabolites (unnamed and named); Figure S3: Principle component analysis of PLHIV-TLE vs. PLHIV-ZLN using the top 30 metabolites. Figure S4: Significant metabolites in lipid metabolism. Fold change indicated changes in PLHIV compared to HC. Supplementary Data file 1: The significant comparisons presented in Figure 2 (Venn diagrams)
