# Supplementary material for: Plasma Metabolic Signature and Abnormalities in HIV-Infected Individuals on Long-Term Successful Antiretroviral Therapy
Source: Metabolites. 2019 Sep 30;9(10):210. doi: 10.3390/metabo9100210 (PMC6835959; doi:10.3390/metabo9100210)
Supplement: Supplementary file 1 [file metabolites-09-00210-s001.zip › metabolites-580629-supplementary 4th/metabolites-580629-Supplementary.pdf]

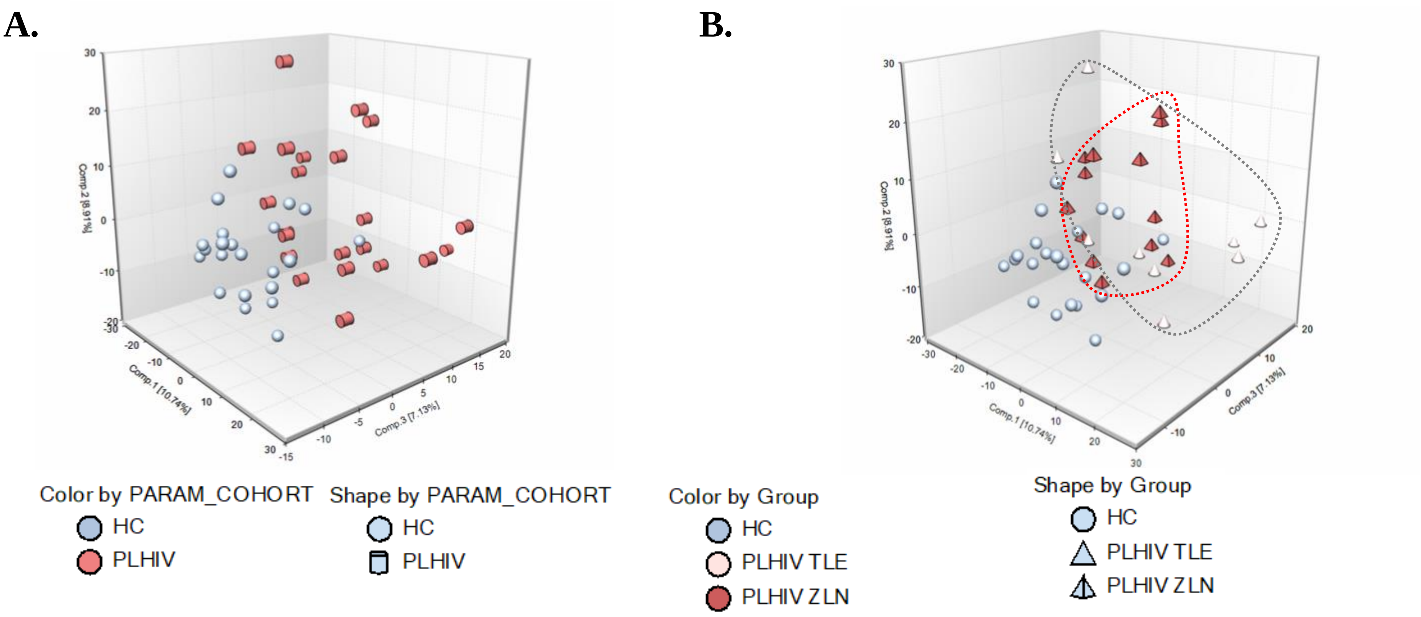

Figure S1. Principal component analysis (PCA). The PCA identified separation between HC and PLHIV samples with PLHIV samples separating to the north and east of HC samples. There was also some separation of samples in the PLHIV group based on treatment type: TDF/3TC/EFV (TLE) and AZT/3TC/NVP (ZLN).

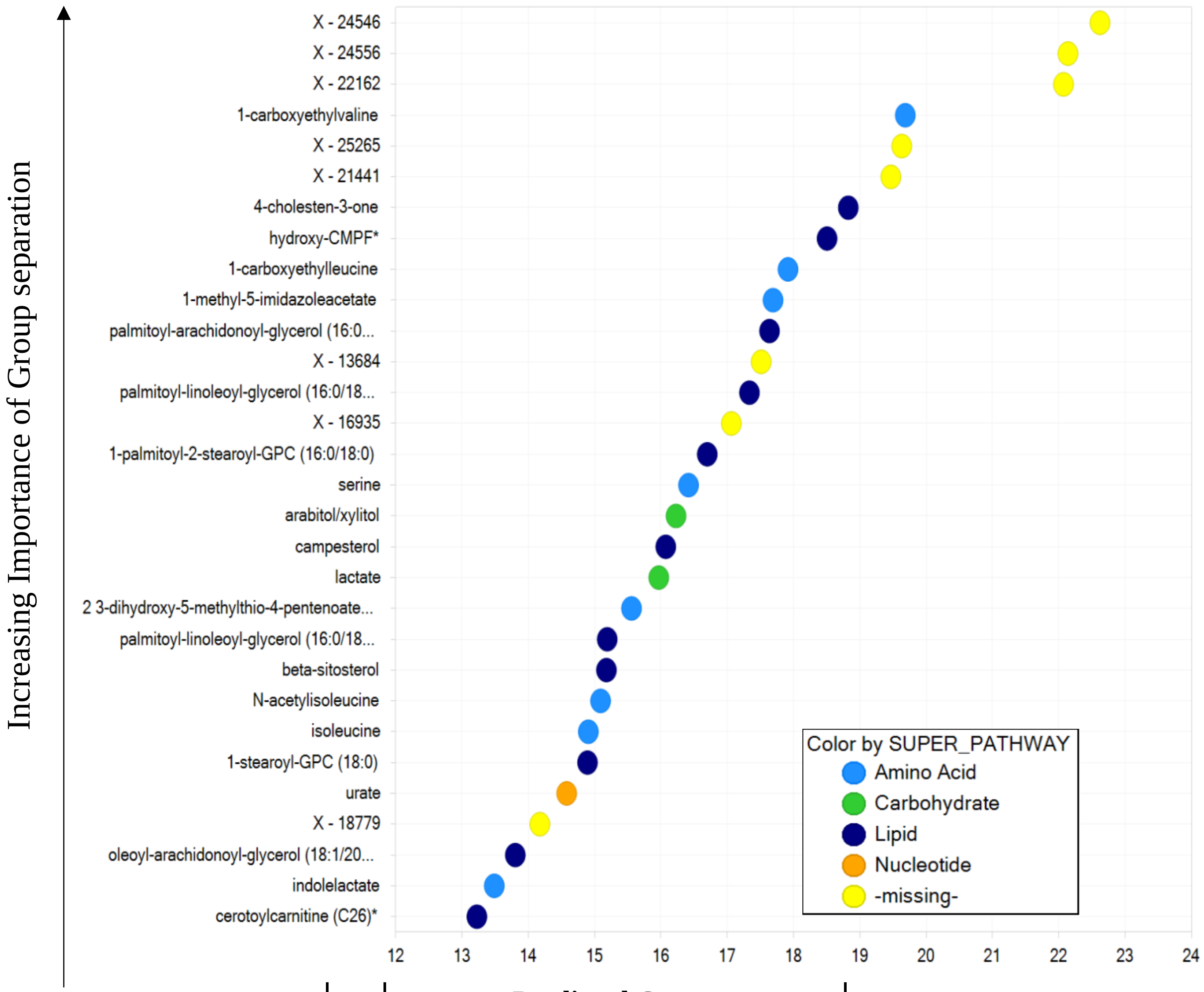

		**Predicted Group**		
**Actual Group**		TLE	ZLN	**Class Error**
	TLE	8	1	0.111
	ZLN	1	12	0.077
	Predictive accuracy = 91%			

Figure S2. Random Forest analysis using all metabolites (unnamed and named)

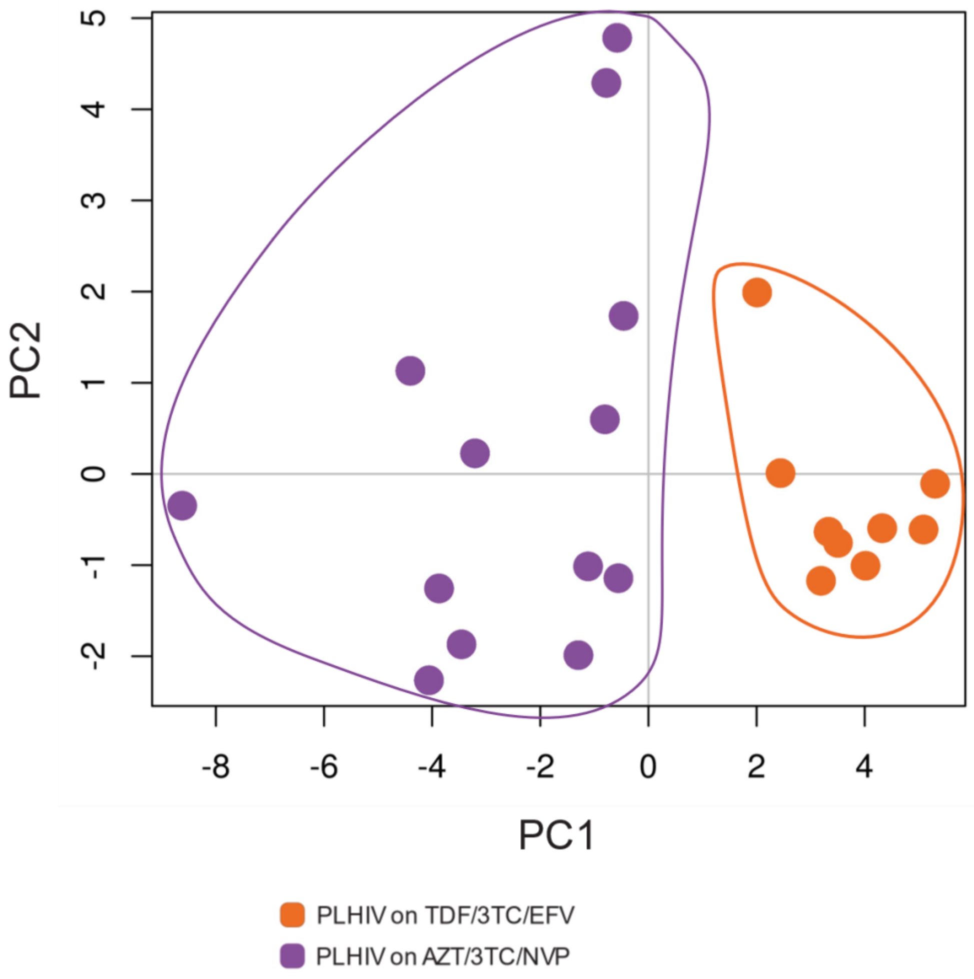

Figure S3. Principle component analysis of PLHIV-TLE vs. PLHIV-ZLN using the top 30 metabolites.

Sub Pathway	Biochemical Name	Fold Change
Long Chain Saturated Fatty Acid	behenate (22:0)*	
Long Chain Polyunsaturated Fatty Acid (n3 and n6)	mead acid (20:3n9)	
Fatty Acid, Dicarboxylate	3-hydroxyadipate*	
	eicosenedioate (C20:1-DC)*	
Fatty Acid Metabolism (Acyl Glycine)	3-hydroxybutyroylglycine**	
Fatty Acid Metabolism (Acyl Carnitine, Long Chain Saturated)	palmitoylcarnitine (C16)	
	stearoylcarnitine (C18)	
	arachidoylcarnitine (C20)*	
	cerotoylcarnitine (C26)*	
Fatty Acid Metabolism (Acyl Carnitine, Monounsaturated)	nervonoylcarnitine (C24:1)*	
	ximenoylcarnitine (C26:1)*	
Fatty Acid Metabolism (Acyl Carnitine, Dicarboxylate)	adipoylcarnitine (C6-DC)	
	pimeloylcarnitine/3-methyladipoylcarnitine (C7-DC)	
Fatty Acid Metabolism (Acyl Carnitine, Hydroxy)	(S)-3-hydroxybutyrylcarnitine	
Fatty Acid, Monohydroxy	2-hydroxyarachidate*	
	3-hydroxyoctanoate	
Fatty Acid, Dihydroxy	2R,3R-dihydroxybutyrate	
Phospholipid Metabolism	choline phosphate	
Phosphatidylcholine (PC)	1-palmitoyl-2-palmitoleoyl-GPC (16:0/16:1)*	
	1-palmitoyl-2-oleoyl-GPC (16:0/18:1)	
	1-palmitoyl-2-linoleoyl-GPC (16:0/18:2)	
	1-palmitoyl-2-dihomo-linolenoyl-GPC (16:0/20:3n3 or 6)*	
	1-stearoyl-2-oleoyl-GPC (18:0/18:1)	
	1-stearoyl-2-arachidonoyl-GPC (18:0/20:4)	
	1,2-dilinoleoyl-GPC (18:2/18:2)	
Phosphatidylethanolamine (PE)	1-palmitoyl-2-linoleoyl-GPE (16:0/18:2)	
	1,2-dilinoleoyl-GPE (18:2/18:2)*	
Phosphatidylinositol (PI)	1-palmitoyl-2-arachidonoyl-GPI (16:0/20:4)*	
	1-palmitoyl-GPA (16:0)	
Lysophospholipid	1-linoleoyl-GPA (18:2)*	
	1-oleoyl-GPC (18:1)	
	1-linolenoyl-GPC (18:3)*	
	1-arachidonoyl-GPC (20:4n6)*	
	1-lignoceroyl-GPC (24:0)	
Plasmalogen	1-(1-enyl-palmitoyl)-2-linoleoyl-GPE (P-16:0/18:2)*	
	1-(1-enyl-palmitoyl)-2-arachidonoyl-GPE (P-16:0/20:4)*	
Diacylglycerol	linoleoyl-linoleoyl-glycerol (18:2/18:2) [2]*	
	stearoyl-arachidonoyl-glycerol (18:0/20:4) [1]*	
	N-palmitoyl-sphingosine (d18:1/16:0)	
Ceramides	N-stearoyl-sphingosine (d18:1/18:0)*	
	N-palmitoyl-sphingadienine (d18:2/16:0)*	
	N-stearoyl-sphingadienine (d18:2/18:0)*	
	ceramide (d18:1/20:0, d16:1/22:0, d20:1/18:0)*	
	ceramide (d18:2/24:1, d18:1/24:2)*	
	myristoyl dihydrosphingomyelin (d18:0/14:0)*	
Dihydrosphingomyelins	palmitoyl dihydrosphingomyelin (d18:0/16:0)*	
	behenoyl dihydrosphingomyelin (d18:0/22:0)*	
	sphingomyelin (d18:0/20:0, d16:0/22:0)*	
Sphingomyelins	hydroxypalmitoyl sphingomyelin (d18:1/16:0(OH))**	
	tricosanoyl sphingomyelin (d18:1/23:0)*	
	sphingomyelin (d18:2/18:1)*	
	sphingomyelin (d18:2/23:1)*	
	sphingomyelin (d18:2/24:2)*	
	sphingomyelin (d17:2/16:0, d18:2/15:0)*	
	sphingomyelin (d18:1/21:0, d17:1/22:0, d16:1/23:0)*	
	sphingomyelin (d18:2/21:0, d16:2/23:0)*	
	sphingomyelin (d18:1/22:2, d18:2/22:1, d16:1/24:2)*	
	sphingomyelin (d18:1/25:0, d19:0/24:1, d20:1/23:0, d19:1/24:0)*	
Sphingosines	hexadecasphingosine (d16:1)*	
Sterol	beta-sitosterol	
	campesterol	
	5alpha-pregnan-3beta,20beta-diol monosulfate (1)	
Progestin Steroids	5alpha-pregnan-3beta,20alpha-diol monosulfate (2)	
	5alpha-pregnan-3beta,20alpha-diol disulfate	
	cortisol	
Androgenic Steroids	16a-hydroxy DHEA 3-sulfate	
	epiandrosterone sulfate	
	androsterone sulfate	
	androstenediol (3alpha, 17alpha) monosulfate (3)	
	5alpha-androstan-3alpha,17beta-diol monosulfate (1)	
	5alpha-androstan-3beta,17beta-diol monosulfate (2)	
	5alpha-androstan-3beta,17beta-diol disulfate	
	andro steroid monosulfate C19H28O6S (1)*	
Primary Bile Acid Metabolism	glyco-alpha-muricholate**	
	glyco-beta-muricholate**	
Secondary Bile Acid Metabolism	glycodeoxycholate	
	taurodeoxycholate	
	glycolithocholate	
	glycoursodeoxycholate	
	hyocholate	
	glycohyocholate	

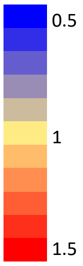

Figure S4. Significant metabolites in lipid metabolism. Fold change indicated changes in PLHIV compared to HC.
